# Strategies for derivation of endothelial lineages from human stem cells

**DOI:** 10.1186/s13287-019-1274-1

**Published:** 2019-07-08

**Authors:** Min Xu, Jiacai He, Chengfei Zhang, Jianguang Xu, Yuanyin Wang

**Affiliations:** 1 Key Laboratory of Oral Diseases Research of Anhui Province, Stomatological Hospital and College, Anhui Medical University, 69 Meishan Road, Hefei, 230032 Anhui Province China; 2 Faculty of Dentistry, The University of Hong Kong, Pokfulam, Hong Kong China

**Keywords:** Endothelial cells, Tissue engineering, Human stem cells, 3D EB formation

## Abstract

Accumulating evidence demonstrates that pre-vascularization of tissue-engineered constructs can significantly enhance their survival and engraftment upon transplantation. Endothelial cells (ECs), the basic component of vasculatures, are indispensable to the entire process of pre-vascularization. However, the source of ECs still poses an issue. Recent studies confirmed that diverse approaches are available in the derivation of ECs for tissue engineering, such as direct isolation of autologous ECs, reprogramming of somatic cells, and induced differentiation of stem cells in typology. Herein, we discussed a variety of human stem cells (i.e., totipotent, pluripotent, multipotent, oligopotent, and unipotent stem cells), which can be induced to differentiate into ECs and reviewed the multifarious approaches for EC generation, such as 3D EB formation for embryonic stem cells (ESCs), stem cell-somatic cell co-culture, and directed endothelial differentiation with growth factors in conventional 2D culture.

## Introduction

Vascular endothelial cells (ECs) constitute the lining of the entire circulatory system. Rapid establishment of blood circulation in post-transplanttissue-engineered constructs is crucial for their initial survival and long-term stability. In particular, pre-vascularization of tissue-engineered constructs as the most promising strategy prior to implantation [1]. ECs are indispensable components in the process of pre-vascularization, exerting a paramount role in vascular functionalities via the interactions with mural cells (smooth muscle cells or pericytes) [2].

Application of autologous ECs represents the most straightforward approach to the pre-vascularization of tissue-engineered constructs. Hagensen et al. [3] isolated primary ECs from immunologically normal mice and subsequently transplanted the ECs into transgenic mice, where the resident primary ECs in the transplanted graft were well integrated and thus contributory to the re-endothelialization of the lesion via migration and proliferation. Nevertheless, the scarce availability of human tissue sources, relatively inefficient expansion due to retarded proliferation, and potential dysfunction of primary ECs from critically ill patients hampered the usage of ECs in clinical applications. Hence, efforts to acquire ECs have focused on stem cell-based approaches. The variety of stem cells, e.g., embryonic stem cells, induced pluripotent stem cells, or adult stem cells, have been explored as sources for EC generation.

**Fig. 1:**
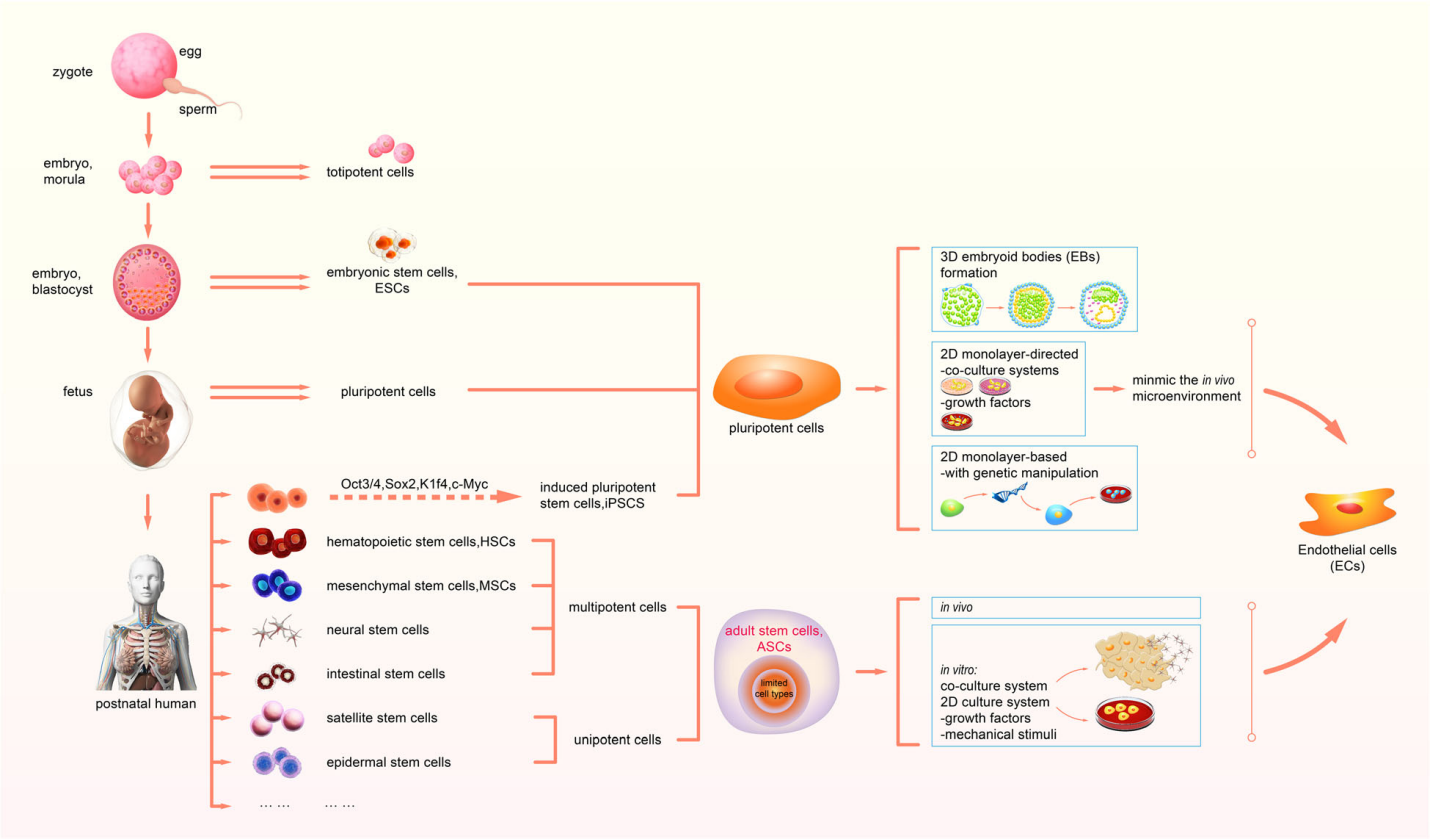
Stem cell-based strategies for human endothelial cell derivation

As per the capacity or potency of differentiation, five types of stem cells are broadly categorized, i.e., totipotent, pluripotent, multipotent, oligopotent, and unipotent [4]. Totipotent stem cells possess the omnipotentiality to differentiate into all cell types, including extra-embryonic lineages, such as cells of the zygotes [5]. The most stringent definition states that the totipotent cells are single cells that can give rise to a new organism for appropriate maternal support, whereas a less stringent definition is that the totipotent cells can generate all the extra-embryonic tissues plus all of the body tissues and the germline [6]. Totipotency was originally experimentally defined, by the experimental criterion, totipotency extends only to the 2C stage in the mouse, or the four- or eight-cell stage in the sheep, cattle, and monkey [7]. Some molecular features of totipotent stem cells have been identified [8, 9], and totipotent cells can be induced to differentiate to endothelium in vitro [10–13]. Pluripotent stem cells retain the potentiality to differentiate into lineages of all three germ layers (i.e., mesoderm, endoderm, and ectoderm), including embryonic stem cells (ESCs) and induced pluripotent stem cells (iPSCs), but cannot generate certain extra-embryonic lineages like trophectoderm (TE) lineages. Pluripotent cells arise subsequent to the establishment of TE lineages by mammalian embryo totipotent cells [6]. Multipotent stem cells can differentiate into confined cell lineages, including bone marrow-derived mesenchymal stem cells (BMMSCs), dental pulp stem cells (DPSCs), and hematopoietic stem cells (HSCs). Oligopotent stem cells exhibit the restricted lineages with the differentiation capacity of a specific tissue, including stem cells residing on the mammalian ocular surface [14]. Unipotent stem cells can differentiate into unilineage, including progenitor cells in postnatal development [15]. Adult stem cells, which exist in the postnatal organism, are either multipotent or unipotent [16], as illustrated by HSCs and mesenchymal stem cells (MSCs) [17]. In this review, we focus on stem cell-based strategies for human endothelial cell derivation (Fig. 1).

## Differentiation of endothelial cells (ECs) from human embryonic stem cells (hESCs) and human-induced pluripotent stem cells (hiPSCs)

ESCs, derived from the inner cell mass (ICM) of blastocyst-stage embryos, are pluripotent stem cells with indefinite self-renewal capacity, usually retaining an undifferentiated status in culture and being capable of differentiation into all the three germ layer lineages under stimuli. The first human ESCs were derived from frozen embryos by in vitro fertilization in 1998 [18]. ESCs have a great potential in human tissue engineering, owing to their pluripotency and capacity to meet “on demand in the laboratory”.

Of note, induced pluripotent stem cells (iPSCs) share high similarity in growth and morphological characteristic to ESCs, as well as the capacity to differentiate to all the cells of three germ layers under stimuli [19, 20]. By the transgenic expression of four transcription factors (TFs) (Oct 3/4, Sox2, Klf4, and c-Myc), mouse fibroblasts can be successfully reprogrammed into iPSCs [21, 22]. As a type of stem cells reprogrammed from terminally differentiated somatic cells, iPSCs serve as a novel cell source for EC generation.

The principal step in initiating in vitro differentiation of ESCs/iPSCs to generate the majority of somatic cell lineages rests on the formation of embryoid bodies (EBs) in suspension culture, which recapitulates the environment of the developing embryo in vivo. Under normal conditions, when cultured in monolayer, ESCs/iPSCs usually produce a multitude of endoderm-like cells. Hence, EB formation has been regarded as a trigger for in vitro differentiation of ESCs/iPSCs. With the generation of EBs, the EC medium is applied to induce the generation of specific EC lineage.

In addition to the formation of EBs [23–32], ESCs/iPSCs can also differentiate into ECs by co-culture with other supporting cells such as OP9 stromal cells [33–36], or culture on the surface of extracellular matrix (ECM) [37–40], or transgenic manipulation such as ETV2 transfection [41, 42].

### Approach of 3D EB formation to derive ECs

EB formation approach is dependent on the spontaneous differentiation of hESCs/hiPSCs in a self-assembledthree-dimensional aggregated structure. Differentiated ECs were isolated by fluorescence-activated cell sorting (FACS) or magnetic-activated cell sorting (MACS) using EC special surface markers, like KDR [24, 43], PECAM1 [23], CD144 [24], or VE-cad [31]. ESCs retain their pluripotency and undifferentiated status when cultured with murine embryonic fibroblast (MEF) feeder layers or in the presence of either leukemia inhibitory factor (LIF) for murine ESCs or basic fibroblast growth factor (bFGF) for human ESCs [44]. Accordingly, the most frequent method for EB generation is to culture hESCs in the absence of MEF feeder layers or bFGF and sustain in suspension culture to avoid cell adherence to the petri dish surface [45].

Levenberg et al. first employed human ESCs to generate ECs [23, 29]. Human ESCs (H9) were cultured in Petri dishes to allow for cell aggregation and prevent cell adherence, so as to facilitate EB formation, wherein H9 cells spontaneously differentiated into a heterogeneous cell population. hESC-derived ECs were isolated by the means of fluorescence-activated cell sorting (FACS) utilizing the EC-specific marker PECAM1. hESCs could spontaneously differentiate into functional ECs independent of stimuli.

Notwithstanding the similarity between hiPSCs and hESCs, doubt arises as to whether hiPSC-derived ECs are equivalent or identical to hESC-derived ECs. Li et al. [30] compared their functionality and gene expression profiles of hiPSC-derived ECs and hESC-derived ECs, by means of induction with EB formation method, respectively. The results showed that hiPSC-ECs and hESC-ECs possessed a similar endothelial gene expression pattern. However, the research demonstrated that hiPSC-derived ECs displayed a low growth rate and rapid loss of endothelial phenotype versus the hESC-derived ECs. The divergence attributed to the variations in gene expression between hiPSCs and hESCs. Another study performed by White et al. [46] also suggested that iPSC- and ESC-derived ECs have similar variance in gene expression. For example, both iPSC- and ESC-derived ECs expressed high levels of KDR and lacked expression of canonical lymphatic-specific genes such as PROX1. Wang et al. [47] demonstrated a high degree of transcriptome similarity between hiPSC- and hESC-ECs. Moreover, Zhao et al. [48] discovered that terminally differentiated ECs derived from iPSCs and ESCs are comparable with regard to transcriptomic aspects provided that they are genetically identical.

Despite the induced differentiation of hESCs/hiPSCs into ECs via EB formation [23–25, 31, 32] (Table 1), low efficiency of EC production resulted due to the uncontrolled spontaneous differentiation in EBs. Therefore, acquirement of high yield of ECs by 3D EB formation has garnered great attention among researchers. Goldman et al. described a short induction protocol in which a high dose of bone morphogenetic protein-4 (BMP4, 50 ng/ml) was employed on day 1 of EB formation (for 1 day only), and by day 12, more than 12% of the total EB cells differentiated into ECs, with a significant increase relative to the report by Levenberg et al. (2% yield) [23, 24] (Table 1). Additionally, another study indicated that the population of ECs in the central region of the EBs was greater than that in the outer region and employment of a “two-step enzyme treatment” cell isolation technique could increase the yield of ECs from EBs (Table 1) [25]. “Two-step enzyme treatment” was used to separate the center and outside the region of EB with trypsin-EDTA and cell dissociation buffer [25]. EB was firstly treated with trypsin-EDTA to detach the outside region, followed by the treatment with cell dissociation buffer to harvest the center region. Aside from these approaches, suppression of the TGF-β pathway [26], the addition of VEGF to differentiation medium [27], hypoxia-induced differentiation [28], and the growth factors or small molecules can also reportedly augment the yield of ECs from EBs [43, 49] (Table 1).

**Table 1:** hESCs and hiPSCs strategies for human endothelial cell derivation

Study	Cell line	Induction system	Purification method	Culture milieu			Induction Period (days)	Efficiency	Positive control	In vitro analysis		In vivo analysis
				Medium	Growth factors	Other special factors				EC markers analyzed	Application/functional assessment	
Levenberg et al. [23]	hESCs:H9	3D EB formation	PECAM1 (FACS)	EGM-2	EGM-2 supplements	No	13–15	2% of total cell population	HUVEC	Genes analysis for PECAM1, VE-cad, CD34, Flk-1, Tie-2 Protein analysis for PECAM1, VE-cad, vWF	In vitro Matrigel sprouting assay LDL uptake assay	Transplantation into SCID mice
Goldman et al. [24]	hESCs:H9	3D EB formation	CD144/KDR (FACS)	knockout DMEM	IL-3, IL-6, G-CSF, Flt-3, SCF	BMP4	12	More than 12% of total cell population	HUVEC	Genes analysis for CD144, KDR Protein analysis for CD144, KDR, CD31, CD34, vWF	In vitro Matrigel sprouting assay Migration assay in response to a VEGF gradient	No
Kim et al. [25]	hESCs: CHA-3;	3D EB formation	No	DMEM+F12 (50% respectively)	No	No	15	–	No	Genes analysis for PECAM1, Flk-1, Tie-2 Protein analysis for PECAM1, vWF	In vitro Matrigel sprouting assay LDL uptake assay	No
Figueiredo et al. [34]	hESCs:H1	2D cells-co-culture system	No	훼-MEM	No	No	9	(CD31+) 18.45% of total cell population	–	Protein analysis for CD31, KDR, CD144	Vascular tube-like structure formation	No
Lippmann et al. [35]	hESCs:H9	2D cells-co-culture system	No	7 days unconditioned medium, 6 days EC medium	–	–	13	(PECAM-1+) 63% of total cell population	No	Genes analysis for PECAM1, vWF, VE-cad Protein analysis for PECAM1, vWF, VE-cad	LDL uptake assay In vitro Matrigel sprouting assay	No
Orlova et al. [37]	hESCs: HES3; HES4	2D growth factor-supplemented differentiation system	CD31 (MACS)	mTeSR1 culture medium	VEGF-A; BMP4; Activin A	CHIR; SB431542	10	(VE-Cad/CD31+) 19.9% of total cell population	No	Protein analysis for CD31, VE-cad, vWF	Tube formation assay	Zebrafish xenotransplantation assay
Kane et al. [40]	hESCs:SA461; SA121	2D growth factor-supplemented differentiation system	No	Large vessel endothelial growth media	Hydrocortisone, EGF, bFGF, heparin	–	14	(VE-Cad/CD31+) 57.04% of total cell population	HUVEC HSVEC HMVEC	Gene analysis for CD31, Flt-1, KDR, VE-cad, CD34 Protein analysis for CD31, VE-cad;	NO production assay Tube formation assay	Transplantation into immunocompromised mice
Elcheva, I. et al. [41]	hESCs:H1; H9	2D genetic manipulation system	No	mTeSR1 culture medium	–	–	5	Not clear	No	Protein analysis for CD31, VE-cad, vWF, CD34, CD73, KDR	LDL uptake assay Tube formation assay	No
Lindgren et al. [42]	hESCs	Genetic manipulation combined with EB formation	No	DMEM+F12	VEGF; bFGF2	–	7	Not clear	No	Gene analysis for CD31, KDR, VE-cad Protein analysis for CD31, KDR, VE-cad	In vitro Matrigel sprouting assay;	No
Adams et al. [31]	hiPSCs	3D EB formation	VE-cad (MACS)	–	–	Fetal calf serum	10	About 18% of total cell population	No	Gene analysis for CD31, KDR, VE-cad Protein analysis for CD31, KDR, VE-cad, eNOS	LDL uptake assay In vitro Matrigel sprouting assay	No
Lin et al. [43]	hiPSCs	3D EB formation with addition of growth factors	KDR (FACS)	EGM2	VEGF bFGF	–	20	About 20% of total cell population	No	Genes analysis for KDR, CD31 Protein analysis for CD31, VE-cad	LDL uptake assay In vitro Matrigel sprouting assay	Matrigel plug assay in NOD/SCID mice
Choi et al. [50]	hiPSCs	2D cell-co-culture system	CD34 (MACS) with CD31 (FCAS)	훼-MEM	–	–	8	2.8%~ 6.0% of total cell population	No	Protein analysis for CD31, KDR, CD49d, CD105, CD144, CD146, vWF, VE-cad	Tube formation assay	No

In general, EB formation permits ESCs/hiPSCs to simultaneously and spontaneously differentiate into all 3 germ layers rather than the desired cell types, which routinely requires a further isolation of the differentiated ECs, thus inevitably resulting in low differentiation efficiency even in combination with additional approaches to enhance differentiation.

### 2D monolayer-directed differentiation approaches

2D monolayer-directed differentiation method refers to the two-dimensional treatment of high-density undifferentiated monolayer stem cells. This strategy usually involves the recruitment of growth factors, such as VEGF, or small molecules.

#### 2D monolayer-directed differentiation approach, with co-culture systems

One of the strategies in inducing hESCs/hiPSCs toward ECs in 2D monolayer-directed differentiation rests on the co-culture of ESCs/hiPSCs with other cell types. OP9 stromal cells, which were derived from mouse bone marrow, are the most prevalent feeder cells employed in co-culture induction system and provide an inductive environment for EC differentiation [33, 34, 50] (Table 1). Vodyanik and Slukvin [33] described a detailed protocol for differentiation of hESCs into ECs using the OP9 co-culture system, isolation of hESC-derived ECs, and analysis of the differentiation efficiency, which was highly reproducible and adopted. Figueiredo et al. [34] (Table 1) performed a proteomic analysis of co-cultured OP9 stromal cells with hESCs and identified that proteins (such as Sparc11) and signaling pathways (such as Nrf2/Nfe212) were involved in this induction protocol. Due to the inevitable drawbacks, such as low differentiation efficiency and susceptibility to contamination with murine stromal cells, this approach is not feasible for the mass generation of ECs.

Lippmann et al. [35] conducted an interesting study (Table 1) in which an “indirect” cell co-culture method was adopted to induce organ-specific ECs. The hESCs were cultured in the unconditioned medium for 3 to 4 days to initiate co-differentiation of neural and endothelial cells, which resulted in large numbers of differentiated cells as immature neuron-like cells with little ECs. The ECs were subsequently co-cultured with “immature neuron-like cells,” and prevailed on days 5 to 7, with blood-brain barrier attributes. The authors speculated that it was the co-induced neural population that provided the organ-specific differentiation cues.

Another study [36] described the generation of brain microvascular endothelial cells (BMECs) via co-culture of hiPSCs and C6 glioma cells in contrast to other types of ECs. BMECs exhibited high expression of tight junction-related genes which are critical to the regulation of the blood-brain barrier. The authors further demonstrated that differentiation was induced by canonical Wnt signals from the C6 rat glioma cell-conditioned medium (C6CM).

#### 2D monolayer-directed differentiation systems, supplemented with growth factors

To date, the monolayer differentiation method supplemented with different growth factors or small molecules at different time points remains to be the most frequent approach in the induction of hESCs/hiPSCs into ECs. The protocol usually divides the whole differentiation process into 2 stages: mesoderm differentiation and the endothelial differentiation, in which the signals involved were manipulated via growth factors/small molecules (i.e., activin A, BMP4, bFGF, CHIR, and BIO) [37, 38, 51–56], some other growth factors (such as VEGF-A, SB431542, and retinoic acid) [26, 39, 49], or their combinations [57] and agonists of signaling pathways [58]. Thus, ECs can efficiently differentiate from hESCs/hiPSCs.

As reported by Orlova et al. [37], differentiation was initiated by the culture of hESCs on a Matrigel-coated surface in mTeSR1 culture medium supplemented with BMP4, VEGF-A (low dose), activin A, and CHIR for 3 days. Subsequently, the mesoderm induction medium was substituted by vascular-specific medium via the removal of mesoderm inductive factors and addition of high doses of VEGF-A and SB431542 (TGF-β signaling inhibitor) for another 6 days, with substantial numbers of ECs generated terminally with high differentiation efficiency (Table 1). Qian et al. [58] documented a facile, chemically defined method to differentiate hiPSCs into BMECs in a developmentally relevant progression via application of CHIR99021 (a canonical Wnt pathway agonist) and a mixture of bFGF, RA, and B27, all of which contribute to the sequential activation of Wnt and RA pathways. Prasain et al. [57] have developed a protocol for the generation of endothelial cells with properties of cord-blood endothelial colony-forming cells (CB-ECFCs) by application of a combination of activin A, BMP-4, FGF-2, and VEGF at a concentration of 10 ng/ml in two-stependothelial-differentiation protocol.

The culture of ESCs requires MEF feeder layers with a basic fibroblast growth factor [59] or Matrigel in the presence of MEF-conditioned medium [60]. Nonetheless, the culture of hESCs on murine feeder layers with MEF-conditioned medium for human therapy is inappropriate whatsoever. Thus, human feeder layers, feeder-free, and completely animal-free conditions have been investigated in many studies [44, 61, 62].

The serum- and feeder-free method to derive functional EC void of EB formation was firstly conducted by Kane et al. [40]. The hESCs cultured in chemically defined “pluripotent maintenance media” could retain a prolonged pluripotency and were subsequently cultured in “endothelial differentiation media” for EC induction. At the end of induction of 14–21 days, hESCs successfully differentiated into ECs with typical “cobblestone” appearance and therapeutic neovascularization function (Table 1).

The researches summarized above showed that hESCs/hiPSCs could be differentiated into ECs under different stimulation. However, whether the differentiated state can be maintained for a long term once the stimulation is suspended still needs further discussion. Lacorre et al. [63] cultured freshly isolated high endothelial venule endothelial cells (HEVECs) for 2 days in normal condition and found that HEVECs rapidly lost their specialized characteristics. Rapid de-differentiation of freshly isolated HEVECs reminds us that even short-term cultures of primary human endothelial cells may lose their identity, not to mention stem cell-derived endothelial cells. In a research performed by Zhao et al. [64], the differentiated ECs maintained their EC identity under continuous stimulation with shear stress or cyclic strain by using bioreactors in vitro or by grafting the vessel into a host organism in vivo.

#### 2D monolayer-based differentiation approach, with genetic manipulation

A more efficient approach to generate ECs from hESCs is the direct manipulation of the expression of TFs, since the Ets (*E*26 transformation-specific sequence) transcription factors are the most preferable reprogramming transgenic genes for EC induction and play a crucial role in the process of vasculogenesis and angiogenesis as well as the regulation of the expression of approximately the whole entity of endothelial specific markers [65].

Elcheva I et al. [41] demonstrated that two groups of TFs (ETV2 combined with GATA2 and TAL1 with GATA2) in 27 candidate factors were capable of directly inducing differentiation of ECs from hESCs. By transduction of ETV2 prior to induction, hESCs acquired the typical morphology and functions of EC (cobblestone) on day 5 of induction (Table 1). Besides the transduction in pre-induction phase, the addition of ETV2 in the induction process was also identified to improve hESC-EC differentiation efficiency. Another study [44] (Table 1) described that transfection of ETV2 on day 4 of differentiation induced 60.4% VE-cadherin-positive ECs, which was significantly higher than the 23.0% obtained in the “before transduction” group.

With respect to iPSCs, some interesting questions have been raised: “Since iPSCs are reprogrammed from the patients’ autogenic cells, can we reprogram ECs into ECs-iPSCs? If so, what about the reprogramming of ECs into iPSCs, followed by differentiation into ECs again?” Haase et al. [66] successfully developed a straightforward method to program human cord blood-derived ECs into iPSCs by transduction of OCT4, SOX2, NANOG, and LIN28. Numerous studies demonstrated that EC derived-iPSCs are differentiated into endothelial lineage better than fibroblast-iPSCs [67–71]. Epigenetic memory inherited from their original tissue have been suggested to influence the differentiation potential of iPSCs [72–74]. In a study performed by Phetfong et al. [75], the methylation levels of endothelial-associated genes from EC-derived iPSCs were lower than those from fibroblast-iPSCs, and the hypomethylation may facilitate these cells to differentiate toward endothelial cell lineage. Furthermore, another study [76] demonstrated a high senescence in vascular lineage cells generated from fibroblast-derived hiPSC. EC-derived iPSCs expanded more robustly and possessed lower rates of senescence and demonstrated more resistance to DNA damage than fibroblast-iPSCs.

Unlike ESCs, patient-specific ECs can be generated from hiPSCs which circumvent the immunological issue. In addition, patient-specific ECs can also be potentially contributed to the study of disease model or drug screening. Gu et al. [77] uncovered the role of BMPR-2 pathway in familial pulmonary arterial hypertension through studying iPSC-ECs.

Moreover, the ethical issue is less ubiquitous for the employment of hiPSCs than the application of hESCs. ESCs are embryo-dependent, while iPSCs avoid the ethical quandaries surrounding embryo destruction [78]. However, there are also some safety concerns in using genetic modification techniques to reprogram somatic cells into iPSCs [79]. To date, extracellular vehicles (EVs) secreted from iPSCs or ESCs are demonstrated with the potential to stimulate angiogenesis, provide cytoprotection, and modulate apoptosis, providing a safer and more effective acellular/cell-free translational therapeutic approach [80].

Nevertheless, the whole procedure of producing hiPSCs-ECs is time-consuming and transgenically expressed transcription factors could be a potential hazard in tumor formation. In general, despite their enormous active impact on the field of EC research, there may be robust ethic concerns by religious communities for the use of hESCs/hiPSCs in scientific studies [81].

## Endothelial cell (EC) differentiation from human adult stem cells (hASCs)

Adult stem cells (ASCs), which are generally tissue-specific and can differentiate into cells of the tissue of origin, exist in fully developed tissues such as the bone marrow, dental pulp, and peripheral blood. In 2002, Jiang et al. [82] revealed that BMMSCs differentiated not only into mesenchymal cells but also cells with endodermal, mesodermal, and ectodermal characteristics under in vitro induction. Further, these cells can contribute to most somatic tissues when engrafted in vivo. Hence, adult stem cells are now categorized into multipotent stem cells which can differentiate into a limited number of cell types and be widely used in tissue engineering.

### Typology of adult stem cells employed in EC differentiation

MSCs and endothelial progenitor cells (EPCs) are two types of adult stem cells that can be potentially utilized for EC generation.

Human MSCs (hMSCs) were first isolated from the bone marrow and have also been isolated from a diverse array of other human tissues thereafter, such as adipose tissue, dental tissues, amniotic fluid and membrane, endometrium, and skin. Numerous studies have successfully induced MSC differentiated into ECs [83–86]. However, the presence of multiple subtypes of MSCs made their endothelial lineage differentiation still debated. A study performed by Fan showed that human bone marrow-derived MSCs did not show an increase in endothelial cell special markers (like CD31, VEGFR2) when cultured in EC differentiation medium [87]. In parallel, Roobrouck et al. [88] demonstrated that the expression of CD31, vWF, and Tie-2 were even decreased when human bone marrow-derived MSCs were treated with VEGF, and these VEGF-treated MSCs also failed in tube formation on Matrigel® assays. Despite this, MSCs are still good candidates in endothelial differentiation.

In general, progenitor cells capable of differentiation into functional ECs are termed as EPCs. With EPCs initially identified and isolated from human peripheral blood by Asahara et al. [89], different progenitor cell populations capable to differentiate into ECs were isolated, such as colony-formingunit-endothelial cells (CFU-EC) [90], colony-formingunit-hill (CFU-Hill) [91], circulating angiogenic cells (CAC) [92], circulating endothelial precursors (CEP) [93], endothelial colony-forming cell (ECFC), low proliferative potential-ECFC (LPP-ECFC), and high proliferative potential-ECFC (HPP-ECFC) [94]. CFU-EC, CFU-Hill, and CAC are categorized as early outgrowth EPC, and ECFCs are classified as late outgrowth EPC [95]. Two distinct approaches are used to acquire EPCs: (a) isolation from blood samples using flow cytometry and (b) in vitro cell culture isolation method [96]. As for flow cytometry isolation, CD34, VEGFR2, and CD133 are often used as markers to isolate EPCs from blood samples. CD34^+^ and CD133^+^ cells were demonstrated to differentiate into endothelial cells [97]; whereas, there is also evidence to the contrary [98]. In terms of the in vitro cell culture isolation method, there is now consensus that two different populations (early and late EPCs) can be distinguished in relation to their culture time [99]. Usually, the late EPCs (also named non-hematopoietic EPCs) possess the ability to differentiate into endothelium [100].

### Approaches to generate endothelial cells from adult stem cells

#### Generation of adult stem cell-derived endothelial cells in vivo

Emerging in vivo approaches for stem cell differentiation have been reported. Liechty et al. [101] performed the first study of a well-defined population of MSCs in prenatal engraftment for in vivo differentiation, in which human MSCs were transplanted into fetal sheep and underwent site-specific differentiation into different cell lineages. Compared with prenatal model systems, postnatal model systems are more feasible to handle and more frequently applied in stem cell-EC differentiation.

Postnatal mouse model of ischemia in a hind limb is most popular for in vivo EC differentiation. In a study by Wu et al. [102], human umbilical cord-derived stem cells were successfully induced into functional ECs and were incorporated with host cells for neovascularization upon transplantation into the ischemic hind limb of nude mice. In addition, the culture of the stem cells in scaffolds and transplantation into the subcutaneous space in the dorsum of nude mice is another popular postnatal model for stem cell-EC differentiation in vivo [103, 104]. When human dental pulp stem cells (hDPSCs) were seeded in scaffolds/tooth slice and then transplanted into the subcutaneous space of nude mice for 4 weeks, the functional blood vessels lined with hDPSC-derived ECs were observed within the scaffolds [103] (Table 2).

**Table 2:** Adult stem cells strategies for human endothelial cell derivation

Study	Cell line	Induction system	Purification method	Culture milieu			Induction period (days)	Efficiency	Positive control	In vitro analysis		In vivo analysis
				Medium	Growth factors	Other special factors				EC markers analyzed	Application/functional assessment	
Zhang et al. [103]	DPSCs	In vivo differentiation system	No	No	No	No	4 weeks	No	HDMEC	Protein analysis for cd31	No	Blood vessel formation in mice
Joddar et al. [83]	BMMSCs	Co-culture system (with fixed ECs/with EC matrix)	No	EBM-2	No	No	7 days	About 70% out of total cells	No	Protein analysis for CD31	No	No
Lozito et al. [84]	BMMSCs	Co-culture system (direct MSC-EC co-culture/indirect MSC-EC co-Culture/with EC matrix)	DiI-labeled FACS sorting	EGM-2-MV	VEGF	–	10 days	No	No	Gene analysis for PECAM Protein analysis for PECAM	Tube formation on Matrigel	No
Gong et al. [107]	SHED	Co-culture system (with EC matrix)	No	EGM-2-MV	VEGF	–	14 days	No	No	Gene analysis for CD31, VEGFR2, VWF	Tube formation on Matrigel	No
Oswald, J. et al. [85]	BMMSCs	Growth factor system	No	DMEM	VEGF	–	7 days	Not clear	No	Protein analysis for KDR, VE-cad, Flt1, vWF	Tube formation on Matrigel	No
Lloyd-Griffith et al. [115]	AFSCs	Growth factor system	No	EndoGro_TM_-VEGF complete media	VEGF hFGF EGF et al.	–	14 days	No	HUVECs	Gene analysis for CD31, VEGFR2, VWF, Angiopoietin 1 Protein analysis for CD31;	In vitro Matrigel sprouting assay LDL uptake assay	No
Xu et al. [116]	SHED/DPSCs	Growth factor system	No	EGM	VEGF	SB-431542	14 days	No	No	Gene analysis for VEGFR1, VEGFR2, CD31, Tie-2, EphrinB2 Protein analysis for CD31, VEGFR2	In vitro Matrigel sprouting assay	Matrigel plug assay
Yuan et al. [86]	hMSCs	Mechanical stimuli system	No	DMEM	bFGF	No	7 days	No	No	Protein analysis for CD31, VE-cadherin, vWF	No	No
Wu et al. [119]	PDMCs	Mechanical stimuli system	No	EGM	VEGF	No	4 days	No	No	Gene analysis for Flt-1, Flk-1, vWF, PECAM-1 Protein analysis for vWF, PECAM-1	LDL uptake assay Tube formation on Matrigel	No

Despite successful stem cell-EC differentiation, the in vivo approach always has methodological flaws such as a plethora of molecular pathways involved in the differentiation process and a large number of lost cells due to local inflammation.

#### Generation of adult stem cell-derived endothelial cells in vitro

##### Differentiation of adult stem cell-derived endothelial cells via co-culture system

Co-culture can simulate an environment similar to native tissues. Adult stem cells could be co-cultured with other cell types within the same culture environment directly or indirectly. In direct co-cultures, adult stem cells are mixed with other cell types and allowed for direct intercellular contact. Intercellular communications occur via three different mechanisms as follows: direct cell junctions, cell-ECM adhesion, and paracrine signaling with soluble factors. In indirect co-cultures, adult stem cells are isolated from other cell types through the cell membrane and cell interactions take place via the secretion of soluble factors, for which transwell culture system is the most common approach.

Direct physical contact between adult stem cells and other cell types can reportedly affect stem cell-EC differentiation [83, 105]. By direct cell-cell contact, signaling pathways such as Notch signaling are activated to induce stem cell differentiation. Joddar et al. [83] (Table 2) reported that in the case of hMSCs cultured onto the chemically fixed ECs, the CD31 expression was pronouncedly upregulated and cobblestone morphology was observed on day 7, implying the differentiation of hMSCs into EC. In addition to contact between different types of cells, the contact with the same type of cells, such as high density of the bone marrow stem cell culture (contact within the compact bone marrow stem cells) has also been identified to trigger stem cell-EC differentiation and further confirmed that the mechanism was mediated by the activated Notch signaling and Notch-inducedVEGF-A [105]. Furthermore, Loibl et al. [106] demonstrated that the direct co-culture of human MSCs and EPCs for 10 days resulted in the differentiation of pericyte-like cells from MSCs and mature ECs from EPCs. The data confirmed that EPCs can differentiate into mature ECs via direct physical contact with MSCs in the absence of any other supplementary growth factors.

Notwithstanding the efficacy of direct cell-cell contact method for EC differentiation, further purification of ECs from a heterogenous co-culture system hinders its widespread application.

The cell-derived ECM constitutes a microenvironment which can provide the stimuli necessary for endothelial differentiation. Cues from the ECM such as topography and mechanical signals play a pivotal role in endothelial differentiation. EC-derived ECM is most common in stimulating stem cell-EC differentiation [84, 105]. In case of hMSCs cultured on the surface of microvascular EC-derived ECM, the expression of PECAM1, an EC-specific surface marker, was observed as well as tube formation on Matrigel [107] (Table 2). Gong et al. reported their analogous results [107] (Table 2) that stem cells from the human exfoliated deciduous teeth were cultivated in the surface of the decellularized extracellular matrix of human umbilical vein endothelial cells for 7 days could express the endothelial-specific surface markers CD31 and VEGFR2, as well as tube formation on Matrigel.

In addition to the methods mentioned above, growth factors derived from the co-culture system have also been reported applied in the induction of endothelial differentiation [84, 108]. Moreover, the mechanism of inducing stem cell differentiation through soluble factors from a co-culture system is comparable to the employment of growth factors within a 2D culture system. However, the differentiation signals usually depend on the origin of the other cell types in the co-culture system [108]. A research by Lozito et al. [84] showed that human MSCs differentiated into the endothelial lineage upon induction by soluble signals from co-cultured ECs.

##### Differentiation of adult stem cell-derived endothelial cells via growth factors within a 2D culture system

Generally, a validated in vitro approach to elicit the differentiation of adult stem cells into ECs would focus on the attempt to recapitulate the process of endothelial differentiation within the developing embryo. By manipulation of the specific endothelial differentiation signaling pathways present in the embryo, the differentiation of stem cells into ECs in vitro may be successfully achieved. The initiation of embryonic endothelial differentiation occurs in the mesoderm in close proximity to the endoderm [109]. Hence, the endoderm-related signals, such as Hedgehog signaling, are crucial in initial EC differentiation [110], while other signaling pathways such as VEGF signaling play a vital role in late EC differentiation. Successful inductions of stem cell-EC differentiation via the modulation of these relevant signaling pathways have been documented in multiple studies.

Among those well-defined signaling pathways, BMP, FGF, and VEGF signaling are the most widely employed inductive cues for in vitro endothelial differentiation [110]. The BMP family, particularly BMP2 and BMP4, modulate early vascular development by triggering the downstream Smad family proteins, as evidenced by gene knockout studies [111, 112]. Notably, the VEGF family members are the first well-described secreted molecules specific to endothelial differentiation. VEGF, through VEGF receptors that are restricted to the endothelial lineage, participate in endothelial differentiation, which may exclude VEGF signaling as the early cues for endothelial differentiation.

Oswald et al. [85] established a BMMSC-EC differentiation protocol based on growth factor VEGF within a 2D culture system (Table 2). Differentiation of ECs was induced by the cultivation of BMMSCs in low-serum (2%) culture medium supplemented with 50 ng/ml VEGF. After induction for 7 days, BMMSCs expressed endothelial-specific markers such as KDR, FLT1, and vWF and possessed the capacity to form capillary structures. Moreover, the endothelial differentiation is significantly enhanced through the regulation of VEGF and VEGF receptor expression. Hypoxia is the most popular approach in promoting EC differentiation via regulated VEGF signaling [28, 113, 114]. The hypoxia-inducible factor 1 (HIF-1), which regulates tissue oxygen tension, is the key factor in enhancing endothelial differentiation in those hypoxic tissues. In a study by Lloyd-Griffith et al. [115] (Table 2), the endothelial differentiation of amniotic fluid-derived stem cells (AFSCs) under the condition of normoxia, intermittent hypoxia, or continuous hypoxia was evaluated, with the results that AFSCs displayed an endothelial-like gene expression profile and functionality when subjected to any conditions above, and hypoxia enhanced the expression of endothelial genes rather than endothelial function.

There are also signaling pathways identified to improve induction efficiency. Xu et al. [116] (Table 2) validated that the differentiation of stem cells from human exfoliated deciduous teeth (SHED) into ECs was evidently augmented via the inhibition of TGF-β signaling. Likewise, a study by Zhang et al. [103] showed that modulation of Wnt/β-catenin signaling in DPSCs also enhanced the efficacy of EC differentiation.

##### Differentiation of adult stem cell-derived endothelial cells utilizing mechanical stimuli within 2D culture systems

Mechanical stimuli such as shear stress can also elicit stem cell-EC differentiation process. Shear stress is the tangential force generated by the flowing blood passing through the endothelial surface of the blood vessels, with a crucial role in the process of both vasculogenesis and angiogenesis in both embryos and adults [117].

Given the essentiality in in vivo endothelial differentiation, it is reasonable to speculate that shear stress might be crucial for in vitro differentiation of ECs. Therefore, a series of models such as the parallel-plate chamber have been employed for investigation of the effect of shear stress on the differentiation of the stem cells into ECs [86, 118–122]. A study documented that subsequent to 2 days of exposure to a two-Pa shear stress and another 5 days of static culture, hMSCs successfully differentiated into ECs in the absence of chemical stimuli [86] (Table 2).

A synergetic relationship between shear stress and growth factors has also been established for stem cell-EC differentiation. Wu et al. [119] (Table 2) cultured the placenta-derived stem cells in EGM (containing growth factors, i.e., VEGF and bFGF) for 3 days and thereafter stimulated with shear stress for 24 h with the finding of successful differentiation of those stem cells into functional ECs.

## Conclusion

In the past decade, diverse types of stem cells and various approaches have been established in EC generation. However, puzzles still remain as to which one is the best, or which one is the worst. ESCs and iPSCs are pluripotent stem cells with the most potent capacity of ECs differentiation and the most convenient characteristics of ‘on demand in the laboratory’. Nevertheless, the time-consuming process of iPSCs production as well as a potential hazard of transgenically-expressed transcription factors in tumor formation for iPSCs and the ethical quandaries surrounding embryo destruction for ESCs impede their application in ECs differentiation. Adult stem cells, despite their differentiation into limited cell types, have drawn relatively higher attention in ECs generation for their wide sources. The accumulating knowledge on stem cells-based endothelial cells differentiation will benefit the quick establishment of blood circulation in those post-transplantedtissue-engineered constructs, which finally improve their initial survival and long-term stability.

## Data Availability

All data generated or analyzed during this study are included in this article.

## Abbreviations

AFSCs: Amniotic fluid-derived stem cells
ASCs: Adult stem cells
bFGF: Basic fibroblast growth factor
BMECs: Brain microvascular endothelial cells
BMMSCs: Bone marrow-derived mesenchymal stem cells
BMP: Bone morphogenetic protein
C6CM: C6 rat glioma cell-conditioned medium
CAC: Circulating angiogenic cells
CB-ECFCs: Cord blood endothelial colony-forming cells
CEP: Circulating endothelial precursors
CFU-EC: Colony-forming unit-endothelial cells
CFU-Hill: Colony-forming unit-hill
CHIR: Small-molecule inhibitor of glycogen synthase kinase-3β
DPSCs: Dental pulp stem cells
EBs: Embryoid bodies
ECFC: Endothelial colony-forming cell
ECM: Extracellular matrix
ECs: Endothelial cells
EPCs: Endothelial progenitor cells
ESCs: Embryonic stem cells
Ets: E26 transformation-specific sequence
FACS: Fluorescence-activated cell sorting
hDPSCs: Human dental pulp stem cells
hESCs: Human embryonic stem cells
HEVECs: High endothelial venule endothelial cells
HIF-1: Hypoxia-inducible factor 1
hiPSCs: Human-induced pluripotent stem cells
hMSCs: Human mesenchymal stem cells
HPP-ECFC: High proliferative potential endothelial colony-forming cell
HSCs: Hematopoietic stem cells
ICM: Inner cell mass
iPSCs: Induced pluripotent stem cells
LIF: Leukemia inhibitory factor
LPP-ECFC: Low proliferative potential endothelial colony-forming cell
MACS: Magnetic-activated cell sorting
MEF: Murine embryonic fibroblasts
MSCs: Mesenchymal stem cells
SHED: Stem cells from human-exfoliated deciduous teeth
TE: Trophectoderm
TFs: Transcription factors
TGF-β: Transforming growth factor-β
VEGF: Vascular endothelial growth factor
